# Impact of *TNFRSF1B* (rs3397, rs1061624 and rs1061622) and *IL6* (rs1800796, rs1800797 and rs1554606) Gene Polymorphisms on Inflammatory Response in Patients with End-Stage Kidney Disease Undergoing Dialysis

**DOI:** 10.3390/biomedicines12061228

**Published:** 2024-05-31

**Authors:** Susana Coimbra, Susana Rocha, Cristina Catarino, Maria João Valente, Petronila Rocha-Pereira, Maria Sameiro-Faria, José Gerardo Oliveira, José Madureira, João Carlos Fernandes, Vasco Miranda, Luís Belo, Elsa Bronze-da-Rocha, Alice Santos-Silva

**Affiliations:** 1 UCIBIO—Applied Molecular Biosciences Unit, Associate Laboratory, Faculdade de Farmácia da Universidade do Porto, 4050-313 Porto, Portugal; srocha@ff.up.pt (S.R.); cristinacatarino@ff.up.pt (C.C.); luisbelo@ff.up.pt (L.B.); elsa.rocha@ff.up.pt (E.B.-d.-R.); 2 Associate Laboratory i4HB—Institute for Health and Bioeconomy, Faculdade de Farmácia da Universidade do Porto, 4050-313 Porto, Portugal; 3 1H-TOXRUN—One Health Toxicology Research Unit, University Institute of Health Sciences, CESPU (Advanced Polytechnic and University Cooperative, CRL), 4585-116 Gandra, Portugal; 4 National Food Institute, Technical University of Denmark, 2800 Kongens Lyngby, Denmark; mjoao.pcv@gmail.com; 5 Health Science Research Centre, University of Beira Interior, 6201-001 Covilhã, Portugal; 6 Hemodialysis Clinic Hospital Agostinho Ribeiro, 4610-106 Felgueiras, Portugal; 7 Hemodialysis Clinic of Porto (CHP), 4200-227 Porto, Portugal; 8 Center for Health Technology and Services Research (CINTESIS), Faculty of Medicine, University of Porto, 4200-450 Porto, Portugal; 9 Hemodialysis Unit of Barcelos | Nefroserve, 4750-110 Barcelos, Portugal; 10 Hemodialysis Unit of Viana do Castelo | Nefroserve, 4900-281 Viana do Castelo, Portugal; 11 Hemodialysis Clinic of Gondomar, 4420-086 Gondomar, Portugal

**Keywords:** SNP, *TNFRSF1B*, *IL6*, inflammatory biomarkers, mortality predictor, ESKD

## Abstract

We aimed to study the impact of polymorphisms in the genes encoding interleukin-6 (IL6) and tumor necrosis factor receptor-2 (TNFR2), reported to be mortality risk predictors, in patients with end-stage kidney disease (ESKD) undergoing dialysis. *TNFRSF1B* (rs3397, rs1061624, and rs1061622) and *IL6* (rs1800796, rs1800797, and rs1554606) polymorphisms were studied in patients with ESKD and controls; the genotype and allele frequencies and the associations with inflammatory and erythropoiesis markers were determined; deaths were recorded throughout the following two years. The genotype and allele frequencies for the *TNFRSF1B* rs3397 polymorphism were different in these patients compared to those in the controls and the global and European populations, and patients with the C allele were less common. Patients with the CC genotype for *TNFRSF1B* rs3397 presented higher hemoglobin and erythrocyte counts and lower TNF-α levels, suggesting a more favorable inflammatory response that seems to be associated with erythropoiesis improvement. Patients with the GG genotype for *TNFRSF1B* rs1061622 showed lower serum ferritin levels. None of the *TNFRSF1B* (rs3397, rs1061624, and rs1061622) or *IL6* (rs1800796, rs1800797, and rs1554606) polymorphisms had a significant impact on the all-cause mortality rate of Portuguese patients with ESKD.

## 1. Introduction

Chronic inflammation is a crucial factor in the development and progression of chronic kidney disease (CKD) and also anemia, the latter of which is common in patients with CKD. Genetic and environmental factors also play a role in an individual’s risk of developing CKD. The presence of genetic variations related to inflammatory biomarkers can thus contribute to kidney disease [1,2]. Moreover, high-throughput genotyping studies and massively parallel sequencing technologies, coupled with the availability of large and diverse datasets containing genomic and health information, have significantly accelerated research in the field of nephrology and have the potential to lead to numerous breakthroughs in understanding and treating kidney-related conditions [3].

Higher levels of soluble tumor necrosis factor receptor 2 (sTNFR2) have been associated with CKD progression and identified as a potential biomarker for the early detection of CKD [4,5]. Moreover, sTNFR2 was reported to be an independent predictor of all-cause mortality in patients with end-stage kidney disease (ESKD) undergoing dialysis [6,7]. Concerning the *TNFRSF1B* gene, the polymorphisms rs3397 (C>T) and rs1061624 (A>G), located in the 3′-untranslated region (3′-UTR), have been studied, and reports suggest that they are able to influence disease outcome by modulating the response to therapy. It has been reported that *TNFRSF1B* rs3397 genotypes have a potential role in the (different) response to infliximab in Japanese patients with Crohn’s disease, an inflammatory bowel condition [8]; in pediatric inflammatory bowel disease, they were associated with subtherapeutic levels of adalimumab [9]; in patients with COVID-19, they influenced the ratio of partial oxygen pressure in arterial blood (PaO_2_) to the fraction of inspiratory oxygen concentration (FiO_2_) [10]. The rs1061624 *TNFRSF1B* polymorphism was reported to be implicated in the development of arterial hypertension in men [11] and associated with long-term response to infliximab in patients with Crohn’s disease [12]. Another single-nucleotide polymorphism (SNP), *TNFRSF1B* rs1061622 (+676 T>G), which results in an amino acid change at position 196 (methionine/arginine), was associated with higher levels of sTNFR2 in inflammatory conditions, such as rheumatoid arthritis [13], systemic lupus erythematosus [14], and preeclampsia [15]. 

Interleukin-6 (IL6), another inflammatory biomarker, is also enhanced in CKD and reported to be an independent predictor of all-cause mortality in patients with ESKD undergoing dialysis [7,16]. The human *IL6* gene is located on chromosome 7p21 and has five exons and four introns, and its promoter region presents some polymorphisms, namely, −174 G>C (rs1800795), −572 G>C (rs1800796), and −597 A>G (rs1800797) [17]. For the *IL6* rs1800795 SNP, we found, in a recent study involving 289 patients with ESKD undergoing dialysis, that the CC genotype presented a trend towards higher levels of inflammatory biomarkers and a higher risk of mortality [18], suggesting that genetic polymorphisms of *IL6* might modulate the inflammatory response and outcome in patients with ESKD and require further research. A study on a large Japanese population reported an association between the *IL6* rs1800796 and kidney function and CKD prevalence [19] and identified this polymorphism as a possible predictor of nephropathy progression in type 2 diabetes patients [20]. *IL6* rs1800797 has also been associated with the glomerular filtration rate and phosphorus and parathyroid hormone levels in patients with CKD [21]. For the intronic SNP *IL6* rs1554606 (+1889 T>G), a trend of more frequent association with microbial keratitis was reported [22]. *IL6* haplotype 222211 (possessing rs2069827, rs1800797, rs1800795, rs1554606, rs2069861, and rs1818879; one codes the common alleles, and two codes the minor alleles) was associated with adiposity [23].

Chronic anemia is common in patients with ESKD [24] and is mainly caused by inadequate renal production of erythropoietin. According to the current guidelines [25], CKD anemia is usually treated with erythropoietic stimulating agents (ESA) and/or iron supplementation. Inflammatory cytokines are known to be key players in anemia associated with chronic inflammatory diseases. For instance, IL6 was reported to compromise mitochondrial function in maturing erythroid cells, impairing hemoglobin production and erythroid maturation [26]. IL6 also stimulates hepcidin expression, which reduces iron absorption and mobilization from iron stores, limiting the iron available for erythropoiesis [27]. Tumor necrosis factor (TNF)-α was found to inhibit erythropoiesis in vivo and in vitro as well as induce a reduction in erythropoietin receptor levels and erythropoietin-induced erythroid progenitor cell proliferation in β-thalassemia/hemoglobin E patients [28].

The hypothesis that polymorphisms of the genes encoding TNFR2 and IL6 are able to modulate the inflammatory response and thereby contribute to the outcome of patients with ESKD by increasing or reducing their mortality risk is an interesting and potentially important area of research that has been poorly studied. The aim of this study was to determine the genotype and allele frequencies of *TNFRSF1B* (rs3397, rs1061624, and rs1061622) and *IL6* (rs1800796, rs1800797, and rs1554606) SNPs in patients with ESKD and controls and evaluate their relationships with circulating levels of inflammatory biomarkers, hemoglobin concentrations and erythrocyte counts, and the outcomes of patients undergoing dialysis.

## 2. Materials and Methods

### 2.1. Subjects

A total of 277 patients with ESKD were recruited from five dialysis clinics in the northern region of Portugal from 2016 to 2019. Adult patients undergoing dialysis treatment for more than 90 days were invited to participate in this study; patients with malignancy, autoimmune disease, or inflammatory or infectious diseases were excluded. A group of 32 healthy volunteers was selected as a control group based on their normal hematological and biochemical values and lack of prior affliction with kidney or inflammatory diseases or anemia; given the age of patients, control subjects were matched, as much as possible, for age and sex with the patient’s group.

At the beginning of this study, demographic, clinical, and dialysis data were registered, and blood was collected for analytical procedures; a follow-up was performed over two years to register events of death. Throughout this follow-up period, 27 patients withdrew from the study, and a total of 51 patients died. This study was conducted in accordance with the Declaration of Helsinki and the Committee on Ethics of the Faculty of Pharmacy, Porto University (Report No. 26-04-2016), and the National Data Protection Commission (Proc. No. 762/2017; Authorization No. 532/2017) approved this study. Patients and controls participated in the study after providing informed and written consent, so their privacy rights were respected.

Therapeutic dialysis was performed 3 times per week for 3–5 h; patients with ESKD were on dialysis treatment for a median period of 3.9 (1.7–7.4) years. Dialysis clearance of urea was expressed as eKt/V.

Demographic, clinical, and analytical data for patients with ESKD and controls at the beginning of the study are presented at Table S1.

### 2.2. Samples

Immediately before a dialysis procedure, blood was collected into tubes with and without anticoagulant (ethylenediaminetetraacetic acid–EDTA) and processed within 2 h of collection to obtain serum, plasma, and buffy coat. Aliquots of the samples were immediately stored at −80 °C until assayed.

### 2.3. Analytical Assays

Hemoglobin concentrations and erythrocyte counts were assessed in whole blood (EDTA) using an automated blood cell counter (Sysmex K1000; Sysmex, Hamburg, Germany).

Serum ferritin and high-sensitivity C-reactive protein (hs-CRP) levels were measured via immunoturbidimetry (Ferritin, Randox Laboratories Ltd., Crumlin, North Ireland, UK; Cardiac C-Reactive Protein (Latex) High-Sensitivity assay, Roche Diagnostics, Basel, Switzerland).

All the other biomarkers were analyzed through enzyme-linked immunosorbent assays (ELISA), according to the manufacturer’s instructions; plasma samples were used to quantify pentraxin (PTX)3 levels (Human Pentraxin 3/TSG-14 Quantikine ELISA Kit, R&D Systems, Minneapolis, MN, USA); in serum, we measured IL6, TNF-α, and sTNFR2 levels (Human IL6 Quantikine HS ELISA Kit, Human TNF-α Quantikine HS ELISA Kit, and Human TNF RII Immunoassay ELISA Kit, R&D Systems, Minneapolis, MN, USA).

Genomic DNA was extracted from buffy-coat samples using a genomic DNA extraction kit (GRiSP, Research Solutions, Porto, Portugal), quantified using NanoDrop-1000 (ThermoFisher Scientific, Wilmington, DE, USA), and analyzed via agarose gel electrophoresis. Trademark TaqMan SNP genotyping assays (Human; ThermoFisher Scientific) were performed to assess the genotype and allele frequencies of *TNFRSF1B* (rs3397, rs1061624, and rs1061622) and *IL6* (rs1800796, rs1800797, and rs1554606) polymorphisms using a real-Time PCR (polymerase chain reaction) system (StepOnePlus, ThermoFisher Scientific).

### 2.4. Statistical Analysis

Statistical analysis was performed using the Statistical Package for Social Sciences (SPSS, version 29, Chicago, IL, USA) for Windows. Shapiro–Wilk analysis was used to determine if data corresponded to a normal distribution. Variables showing a Gaussian distribution are presented as means ± standard deviations (SD), and those non-normally distributed are presented as medians [interquartile ranges]. For categorical variables, the comparison between groups was analyzed using the Chi-squared test or the Fisher exact test. Odds ratio (OR) was calculated to assess the association between the type of allele and the type of individual regarding affliction with CKD or lack thereof. For continuous variables, differences between groups were evaluated using Mann–Whitney *U* test, independent *t*-test, or the one-way ANOVA test (supplemented with Bonferroni post hoc after transforming variables (when necessary) to achieve normal distribution). The strength of the correlations between variables was determined through the Spearman’s rank correlation coefficient. Survival distribution comparisons between genotypes were performed using the log-rank test. The Cox Proportional Hazards Survival Regression model with confounding variables (age, dialysis vintage, vascular access, type 2 diabetes, and cardiovascular disease) was used to estimate, in the follow-up period of two-years, the all-cause mortality hazard ratio (HR) according to the polymorphic genotypes. A value for which *p* < 0.05 was considered statistically significant.

## 3. Results

### 3.1. General Demographic, Clinical, and Analytical Data

Patients with ESKD presented, compared to the controls, lower values of hemoglobin concentrations and erythrocyte counts and higher levels of all the inflammatory biomarkers studied, with IL6, TNF-α, and sTNFR2 showing the highest increases (Table S1).

### 3.2. Genotype and Allele Frequencies for Each SNP Studied

For the SNP *TNFRSF1B* rs3397 (Table 1), the genotype and allele frequencies between patients and controls were different; for *TNFRSF1B* rs1061624 and rs1061622 polymorphisms (Table 1 and Table S2), no significant differences were found. Additionally, for *TNFRSF1B* rs3397, a significant OR (1.911, 95%CI [1.133–3.233]) regarding the likelihood of patients being allele T carriers in relation to the controls was observed, while for the other *TNFRSF1B* SNPs, no significant associations (OR) were found (Table S2).

Regarding *IL6* SNPs (Table 2), the genotype and the allele frequencies between patients with ESKD undergoing dialysis and controls were similar for rs1800796, rs1800797 and rs1554606, as well as no significant OR (Table S2) were found.

### 3.3. Impact of TNFRSF1B and IL6 SNPs on Inflammatory Biomarkers, Hemoglobin Concentrations, and Erythrocyte Count

Regarding sTNFR2 levels (Table 3), no significant differences were observed between patients with the different genotypes of the *TNFRSF1B* SNPs studied.

For the SNP *TNFRSF1B* rs3397 (Table 3), patients with the CC genotype, compared to the TT and TC genotype carriers, presented higher hemoglobin concentrations and erythrocyte counts and lower levels of TNF-α; these patients with the CC genotype also showed lower TNF-α/sTNFR2 ratios than the TT genotype patients; moreover, although without statistical significance, the other inflammatory parameters studied showed the lowest median values in patients with the CC genotype.

Given the different values of hemoglobin and erythrocyte counts for patients with the *TNFRSF1B* rs3397 SNP (Table 3), we analyzed their need to receive iron and ESA therapies to treat anemia, according to the genotypes. Among those with the CC genotype (*n* = 26), 15 (57.7%) patients were adhering to an iron supplementation regimen (25 [25–50] mg/week), and 20 (76.9%) were undergoing ESA (0.35 [0.20–0.53] μg/kg/week) treatment; for TT carriers (*n* = 128), 83 (64.8%) patients were medicated with iron (50 [25–60] mg/week), and 104 (81.3%) were treated with ESA (0.38 [0.24–0.72] μg/kg/week); regarding the TC genotype patients (*n* = 123), 77 (62.6%) were undergoing iron supplementation treatment (40 [25–80] mg/week), and 105 (85.4%) were undergoing ESA treatment (0.36 [0.18–0.56] μg/kg/week). The dose of iron used for the TT carriers’ treatment was significantly higher than that used for CC carriers (*p* = 0.045); moreover, the dose of ESA used to treat TT carriers was significantly higher than that used to treat the TC genotype patients (*p* = 0.012).

For the *TNFRSF1B* rs1061624 SNP (Table 3), the patients with the AG genotype were younger than the GG genotype patients and had a shorter dialysis vintage than the AA genotype carriers; AA genotype patients showed significantly higher erythrocyte counts than the GG genotype carriers.

Regarding the *TNFRSF1B* rs1061622 SNP (Table 3), the GG genotype patients, compared to TT and TG genotype carriers, presented significantly lower serum ferritin levels; TG genotype patients showed higher values of TNF-α and TNF-α/sTNFR2 than TT genotype subjects. Considering the lower value of serum ferritin in this polymorphism, we analyzed the number of patients that were undergoing ESA and iron therapies, according to genotypes, and found that the number of patients needing ESA and iron treatments was similar among the different genotypes (χ^2^; *p* = 0.771, *p* = 0.292, and *p* = 0.716; *p* = 1.000, *p* = 0.407, and *p* = 0.598; respectively).

Regarding the *IL6* rs1800796 SNP (Table 4), no significant differences were observed.

For the *IL6* rs1800797 SNP (Table 4), AG genotype carriers presented significantly lower TNF-α levels compared to those with the GG genotype.

Regarding the *IL6* rs1554606 SNP (Table 4), the TG genotype carriers, compared to the GG genotype carriers, presented significantly higher hs-CRP values.

Considering the data found for *TNFR2* rs3397 polymorphism, we evaluated the correlations between the studied inflammatory biomarkers within the *TNFRSF1B* rs3397 polymorphic genotypes in patients with ESKD (Table 5).

### 3.4. Impact of TNFRSF1B and IL6 SPNs on All-Cause Mortality of Patients with ESKD

As already mentioned, throughout the two-year follow-up, to register events of death, 27 patients left the study. A total of 51 patients were reported to have died over the two-year follow-up period due to different causes, including cardiovascular events, cachexia, infectious diseases, or others. Considering the *TNFRSF1B* (rs3397, rs1061624, and rs1061622) and the *IL6* (rs1800796, rs1800797, and rs1554606) polymorphisms, the frequencies of the different genotypes were similar for patients who were alive after the two-year follow-up and for those who died during this follow-up period (χ^2^: *p* = 0.143; *p* = 0.261 and *p* = 0.778 as well as *p* = 0.597, *p* = 0.495, and *p* = 0.389, respectively) (Table S3).

No significant differences in the cumulative survival curves of the different genotypes were observed for *TNFRSF1B* rs3397 (*p* = 0.433), rs1061624 (*p* = 0.172), or rs1061622 (*p* = 0.840) or for *IL6* rs1800796 (*p* = 0.578), rs1800797 (*p* = 0.346), or rs1554606 (*p* = 0.258) (Figure S1). Additionally, the all-cause mortality HR between the different polymorphic genotypes, accounting for age, dialysis vintage, vascular access, type 2 diabetes, and cardiovascular disease as biases, showed no statistically significant differences in the two-year follow-up period; the corresponding results are as follows: (1) *TNFRSF1B* rs3397 (*p* = 0.884), rs1061624 (*p* = 0.141), and rs1061622 (*p* = 0.563), and (2) *IL6* rs1800796 (*p* = 0.374), rs1800797 (*p* = 0.908), and rs1554606 (*p* = 0.855) (Table S4).

## 4. Discussion

In this study, we evaluated and compared the distribution of genetic variants in *IL6* and *TNFRSF1B* genes in patients undergoing dialysis and in controls. Additionally, we studied the association of the SNPs with anemia, inflammation, and all-cause mortality risk.

Regarding the *TNFRSF1B* rs3397 SNP, the genotype and allele frequencies were significantly different in patients and controls despite the low number of participants in the latter group; to minimize this limitation, we supported the findings by comparing our data with global and European populations. Considering the Reference SNP (rs) Report database [29] for rs3397, the allele frequencies found for our study groups, compared to those reported for global (C: 0.417; T: 0.583) and European (C: 0.363; T: 0.637) populations, were similar for the controls (*p* = 0.401 and *p* = 0.079, respectively) but different for patients with ESKD (*p* < 0.001 and *p* = 0.021, respectively). According to our data, it seems that in the ESKD population, the C allele is less frequent than in our controls and the reference values from global and European populations. In fact, we found that our patients were 1.9× more likely to be allele T carriers than the controls (OR, Table S2). For other inflammatory conditions, the allele frequencies reported did not differ from those presented by the control groups [10,30,31]. As far as we know, no data have been reported concerning *TNFRSF1B* rs3397 SNP frequencies in a CKD cohort.

Regarding the influence of *TNFRSF1B* rs3397 polymorphism on sTNFR2 levels, there are also few data in the literature. It was reported that it is not a major contributory factor for the genetic risk of type 2 diabetes [31] and appears to affect the response to biological therapies, used to treat some inflammatory disorders [8,9]. TNFR1 is known to limit the effects of TNF-α, reducing the inflammatory response, by linking circulating TNF-α, neutralizing the available TNFRs on cell surfaces and decreasing their numbers [32]. The levels of sTNFRs are higher in inflammatory conditions, and, as carriers of TNF-α, sTNFRs are able to mediate inflammatory activation at different organs, eventually augmenting TNF-α’s effects by prolonging its function [33]; furthermore, they can also be used as biomarkers of disease severity. TNFR2, expressed on immune cells, endothelial cells, mesenchymal stem cells, and neural cells, interacts with TNF-α, leading to cell proliferation and survival. The TNF-α/TNFR2 signaling pathway is considered to be important for controlling the immunoregulatory functions of almost all TNFR2^+^ cells [34]. In patients with COVID-19, *TNFRSF1B* rs3397 genotypes did not influence the levels of sTNFR2 and TNF-α [10].

Besides the different genotype distributions for the *TNFRSF1B* rs3397 SNP between the patients with ESKD and controls, we also found that CC patients presented a lower-level inflammation status, namely, lower levels of TNF-α and lower TNF-α/sTNFR2 ratios. IL6, PTX3, and ferritin levels showed a trend towards lower values in this group of patients, but without statistical significance (Table 3). This reduced inflammation status may partially explain the lower severity of anemia in the CC patients, as it is known that increased levels of inflammatory cytokines, such as TNF-α and *IL6*, can disturb erythropoiesis. It is worth highlighting that this group presented higher levels of hemoglobin, which were not associated with higher therapeutic doses of iron and/or ESA. Despite the lower degree of inflammation in the CC group, we did not find a lower mortality rate among these patients at the 2-year follow-up.

For the other *TNFRSF1B* polymorphisms (rs1061624 and rs1061622), the genotype and allele frequencies in patients undergoing dialysis and controls were similar. Regarding the *TNFRSF1B* rs1061624 SNP, and when comparing our results for with those for the global (A: 0.459; G: 0.541) and European (A: 0.445; G: 0.555) populations [29], no differences in the allele frequencies were found for the controls (*p* = 0.730; *p* = 0.904, respectively) and patients (*p* = 0.652; *p* = 0.833, respectively). An analogous pattern was observed for *TNFRSF1B* rs1061622 polymorphism, for which the allele frequencies in the global (T: 0.769; G: 0.231) and European (T: 0.766; G: 0.234) populations [29] were similar to those presented by our controls (*p* = 0.156; *p* = 0.142; respectively) and patients (*p* = 0.616; *p* = 0.505; respectively).

The *TNFRSF1B* rs1061624 polymorphism is associated with the development of arterial hypertension in men [11], long-term response to infliximab in individuals with Crohn’s disease [12], and an increased risk of metabolic syndrome [35]. It was also reported to influence the circulating levels of TNF-α in the Tatar ethnic group [35]. According to our data, the *TNFRSF1B* rs1061624 SNP does not significantly influence the levels of the studied markers or impact the mortality rate of Portuguese patients with ESKD undergoing dialysis.

The *TNFRSF1B* rs1061622 SNP has been associated with some inflammatory disorders [10,14,15]. In the case of psoriasis, an inflammatory dermatologic disease, the *TNFRSF1B* rs1061622 SNP was reported to be associated with the risk of developing this disease and with the response to psoriasis treatment with anti-TNF or anti-IL12/IL23 agents [36]; in patients with COVID-19, the GG carriers presented significantly lower levels of sTNFR1 and a trend towards lower values of sTNFR2 compared to those with the TT and TG genotypes [10]; subjects with rheumatoid arthritis did not present differences in sTNFR2 concentrations between *TNFRSF1B* rs1061622 SNP genotypes [13]. In the present work, this polymorphism did not affect sTNFR2 or TNF-α/sTNFR2 values and did not influence the mortality rate. However, we found that the GG genotype patients for *TNFRSF1B* rs1061622 polymorphism showed decreased levels of serum ferritin compared to TT and TG genotype carriers. We did not find differences in hemoglobin concentration or erythrocyte count between patients with the different genotypes for this SNP, and the quantities of patients receiving iron and/or ESA therapies were also similar in the three genotype groups. As mentioned, sTNFR levels have been associated with concentrations of inflammatory biomarkers, such as IL6 and ferritin, and with the development of acute kidney injury in patients with COVID-19 admitted to intensive care units [31]. We could hypothesize that the decreased values of ferritin, found for the GG genotype, could be associated with lower inflammatory activity; however, we did not find differences between genotypes of the *TNFR2* rs1061622 polymorphism in the other studied inflammatory biomarkers. Further studies are required to better understand how the *TNFR2* rs1061622 SNP influences serum ferritin levels.

It was reported that the three SNPs in the promoter region of *IL6* present ethnic disparities, as shown via a comparison of allele frequencies in different populations worldwide; rs1800797 was found to be highly polymorphic in Caucasians but almost monomorphic in Asians and Africans, while rs1800796 variation was highest in East Asians, followed by South Asians and Americans, but it was rare in Africans and Europeans [37]. In the present work, for the *IL6* rs1800796 polymorphism, we found no differences for the allele frequencies in the controls and patients with ESKD; and, when comparing these values with those for the global (G: 0.929; C: 0.071) and European (G: 0.950; C: 0.050) populations [29], the allele frequencies are similar to those found for the controls (*p* = 0.791; *p* = 0.646; respectively) and patients (*p* = 0.378; *p* = 0.219; respectively). Concerning the *IL6* rs1800797 and rs1554606 SNPs, we did not find significant differences between the genotype and allele frequencies in patients and controls. However, in the *IL6* rs1800797 SNP, for the controls, we found a trend towards higher (*p* = 0.105) and significantly different (*p* = 0.040) allele frequencies when compared with the reference values [29] for the global population (A: 0.363; G: 0.637) and the European population (A: 0.391; G: 0.609), respectively. In *IL6* rs1554606, for controls, we observed a trend towards different allele frequencies (*p* = 0.090; *p* = 0.050) when compared with the reference values [29] for the global population (T: 0.417; G: 0.583) and for the European population (T: 0.434; G: 0.566). In the case of patients with ESKD, the allele frequencies of both *IL6* rs1800797 (*p* < 0.001; *p* < 0.001; respectively) and *IL6* rs1554606 SNPs (*p* = 0.013; *p* < 0.001; respectively) differed significantly from those of both the global and European populations [29]. According to a meta-analysis conducted to determine the effect of *IL6* promoter polymorphism (−174 G>C, −572 G>C, and −597 G>A) on the development of rheumatoid arthritis stratified by ethnicity, an ethnic sub-analysis of data revealed that genetic differences within a country can vary significantly [38]. The individual genotype, much more than the ethnic or geographic affiliation, should be considered; a distinction could be made between population-specific polymorphisms and continent- or race-specific genetic polymorphisms [39].

Concerning the *IL6* gene, the more prevalent haplotype in East Asia (C–T–T, represented by variant alleles of rs1800796, rs1524107, and rs2066992) was associated with lower IL6 levels, while higher expression of IL6 was observed in the European population, in which rs1800797 and rs1800795 were highly polymorphic [37]. *IL6* rs1800796 SNP was associated with kidney function and CKD prevalence in a large Japanese population [19]. Ali et al. reported that the *IL6* −597 G/A (rs1800797) as well as the −174 (rs1800795) G/C polymorphisms may play a role in susceptibility to hepatitis C virus infection among Egyptian hemodialysis patients [40]. *A* meta-analysis revealed that *IL6* rs1800795, rs1800796, and rs1800797 SNPs are important players in diabetic nephropathy development [41]. Among elderly Brazilian patients with hip and knee osteoarthritis, individuals with the GC and CC genotypes of the *IL6* rs1800796 polymorphism were found to have lower IL6 levels compared to those with the GG genotype [42]. For the *IL6* rs1800796 SNP, Japanese patients with type 2 diabetes carrying the G allele had a greater IL6 secretion capacity than those without the *G allele [43]. The *IL6* rs1800797 GG genotype was found to be associated with higher IL6 levels in patients with pulmonary chronic obstructive disease from Romania [44] as well as in type 2 diabetic patients from Indonesia [45]. A study from Brazil reported that the *IL6* −597 G>A (rs1800797) polymorphism did not influence IL6 levels in either individuals with Down syndrome or controls [46]. Concerning *IL6* rs1554606, data are scarce; it has been associated with microbial keratitis cases in a South Indian population [22] and was reported to integrate an *IL6* haplotype, along with rs2069827, rs1800797, rs1800795, rs2069861, and rs1818879, that was associated with adiposity [23]. According to our data, in Portuguese patients with ESKD undergoing dialysis, the *IL6* rs1800797, rs1800796, and rs1554606 polymorphisms do not seem to have a significant influence on the levels of IL6 or the values of the other inflammatory biomarkers studied. However, the patients with the CC genotype of the *IL6* SNP rs1800796 showed a trend towards lower IL6 levels, suggesting that this polymorphism may modulate IL6 levels; indeed, the small sample size of the CC genotype carriers in our cohort limits our conclusions and thereby warrants further analysis using a larger population of patients with ESKD to clarify the data.

The sample size of the control group, for the reasons already mentioned, and of some of the subgroups of patients should have been larger, and this may represent a limitation of this study. Accordingly, in order to overcome this constraint and increase the confidence of our data, we compared the genotype distributions obtained for our Portuguese patients and control cohorts with those described for the European and global populations.

## 5. Conclusions

In summary, the allele frequencies of the *TNFRSF1B* rs3397 SNP were different in patients with ESKD and controls, with the C allele showing a lower frequency when compared to the controls and global or European populations. In patients, the CC genotype for the rs3397 polymorphism was associated with decreased levels of inflammation and a less severe anemic condition, suggesting a more favorable inflammatory response that may also contribute to improving erythropoiesis. For *IL6* SNPs rs1800797 and rs1554606, the allele frequencies for the patients and controls, although not distinct from each other, differed significantly from the values described for global and European populations; for the other polymorphisms studied, no differences were found in the allele frequencies presented by the controls and patients (and the frequencies were similar to those described for the global and European populations). The *TNFRSF1B* rs1061624 and the *IL6* rs1800797/rs1800796/rs1554606 polymorphisms did not show substantial influences on the levels of the analyzed biomarkers. However, the association of *TNFRSF1B* rs1061622 with serum ferritin levels, and the possible modulation of IL6 levels by the *IL6* rs1800796 SNP, require additional studies to confirm and explain the data. None of the *TNFRSF1B* (rs3397, rs1061624, and rs1061622) or *IL6* (rs1800796, rs1800797 and rs1554606) polymorphisms studied had an impact on the mortality rate of Portuguese patients with ESKD.

## Figures and Tables

**Table 1:** Genotype and allele frequencies of *TNFRSF1B* (rs3397, rs1061624, and rs1061622) single-nucleotide polymorphisms in control groups (*n* = 32) and patients with end-stage kidney disease (ESKD; *n* = 277).

	Control	ESKD	*p* (χ^2^)
**rs3397**			
**Genotype (*n*, %)**	**Frequency**		
CC	7; 21.9%	26; 9.4%	0.039
TT	9; 28.1%	128; 46.2%	
CT	16; 50.0%	123; 44.4%	
**Allele**	**Frequency**		
C	0.47	0.32	0.014
T	0.53	0.68	
**rs1061624**			
**Genotype (*n*, %)**	**Frequency**		
AA	6; 18.8%	56; 20.2%	0.980
GG	10; 31.3%	84; 30.3%	
AG	16; 50.0%	137; 49.5%	
**Allele**	**Frequency**		
A	0.44	0.45	0.855
G	0.56	0.55	
**rs1061622**			
**Genotype (*n*, %)**	**Frequency**		
TT	23; 71.9%	174; 62.8%	0.518
GG	1; 3.1%	20; 7.2%	
TG	8; 25.0%	83; 30.0%	
**Allele**	**Frequency**		
T	0.84	0.78	0.225
G	0.16	0.22	

χ^2^ Pearson’s Chi-squared test.

**Table 2:** Genotype and allele frequencies of *IL6* (rs1800796, rs1800797, and rs1554606) single nucleotide polymorphisms in control group (*n* = 32) and patients with end-stage kidney disease (ESKD; *n* = 277).

	Control	ESKD	*p* (χ^2^)
**rs1800796**			
**Genotype (*n*, %)**	**Frequency**		
GG	29; 90.6%	247; 89.2%	0.664
CC	1; 3.1%	4; 1.4%	
GC	2; 6.3%	26; 9.4%	
**Allele**	**Frequency**		
G	0.94	0.94	0.972
C	0.06	0.06	
**rs1800797**			
**Genotype (*n*, %)**	**Frequency**		
AA	3; 9.4%	31; 11.2%	0.723
GG	18; 56.3%	135; 48.7%	
AG	11; 34.4%	111; 40.1%	
**Allele**	**Frequency**		
A	0.27	0.31	0.444
G	0.73	0.69	
**rs1554606**			
**Genotype (*n*, %)**	**Frequency**		
TT	4; 12.5%	34; 12.3%	0.887
GG	16; 50.0%	127; 45.8%	
TG	12; 37.5%	116; 41.9%	
**Allele**	**Frequency**		
T	0.31	0.33	0.752
G	0.69	0.67	

χ^2^ Pearson’s Chi-squared test.

**Table 3:** Age, sex, dialysis vintage, and analytical data according to the *TNFRSF1B* (rs3397, rs1061624 and rs1061622) polymorphic genotypes for patients with end-stage kidney disease.

	Age (Years)	Sex, F/M (%)	DV (years)	Erythrocyte (×10^12^/L)	Hb (g/dL)	Ferritin (ng/mL)	hs-CRP (mg/dL)	IL6 (pg/mL)	TNF-α (pg/mL)	sTNFR2 (ng/mL)	TNF/TNFR2 (×10^−3^)	PTX3 (ng/mL)
**rs3397**												
CC (*n* = 26)	67 ± 15	42/58	5.33 [2.14–7.21]	3.95 [3.69–4.29]	12.1 [11.3–12.7]	274 [151–400]	0.27 [0.13–0.74]	3.78 [2.22–5.00]	2.90 [2.55–3.33]	14.6 [11.8–16.2]	0.20 [0.17–0.25]	1.05 [0.75–1.75]
TT (*n* = 128)	70 ± 12	44/56	3.55 [1.49–7.61]	3.74 [3.44–4.00] **	11.6 [10.6–12.3] *	323 [184–476]	0.42 [0.18–0.88] ^a^	4,57 [2.73–7.64] ^b^	3.57 [2.72–4.76] **	14.3 [11.6–17.4]	0.25 [0.19–0.37] **	1.41 [1.03–2.06] ^e^
TC (*n* = 123)	68 ± 15	44/56	3.87 [1.68–6.71]	3.70 [3.40–3.98] **	11.3 [10.6–12.0] **	299 [174–458]	0.37 [0.14–0.80]	4,27 [2.92–7.02] ^c^	3.45 [2.69–4.76] **	15.1 [12.3–17.9]	0.23 [0.18–0.31] ^d^	1.43 [1.00–2.09] ^f^
**rs1061624**												
AA (*n* = 56)	69 ± 14	45/55	5.74 [2.42–8.01]	3.81 [3.51–4.04] ^λ^	11.7 [11.0–12.4] ^g^	326 [180–465]	0.30 [0.13–0.81]	3.78 [2.25–6.26]	3.05 [2.65–4.03] ^h^	14.2 [11.6–16.7]	0.25 [0.18–0.29]	1.38 [0.94–2.08]
GG (*n* = 84)	71 ± 14	42/58	4.16 [1.82–7.81]	3.66 [3.36–4.04]	11.4 [10.5–12.1]	285 [178–454]	0.37 [0.15–0.77]	3.88 [2.90–7.15]	3.59 [2.68–5.08]	14.7 [11.6–17.8]	0.24 [0.20–0.35]	1.28 [0.94–1.83]
AG (*n* = 137)	67 ± 14 ^λ^	46/54	3.22 [1.41–6.59] ^κ^	3.72 [3.50–4.00]	11.5 [10.7–12.3]	334 [178–455]	0.42 [0.18–0.87]	4.70 [2.78–7.50] ^i^	3.39 [2.70–4.63]	14.8 [12.0–17.9]	0.22 [0.18–0.32]	1.53 [1.07–2.16] ^j^
**rs1061622**												
TT (*n* = 174)	68 ± 14	48/52	4.09 [2.01–7.57]	3.80 [3.48–4.01]	11.5 [10.7–12.3]	321 [178–475] ^Ψ^	0.39 [0.18–0.81]	4.03 [2.68–6.83]	3.30 [2.61–4.53]	14.7 [11.9–18.0]	0.22 [0.17–0.31]	1.41 [0.98–2.10]
GG (*n* = 20)	67 ± 13	50/50	5.68 [1.95–8.46]	3.88 [3.54–4.07]	11.6 [10.8–12.7]	182 [135–366]	0.38 [0.20–1.11]	4.46 [3.91–7.03]	3.78 [2.82–5.01]	15.3 [12.9–17.3]	0.25 [0.20–0.30]	1.24 [0.94–1.85]
TG (*n* = 83)	70 ± 13	36/64	2.70 [1.34–6.27] ^δ^	3.70 [3.37–3.96]	11.3 [10.6–12.2]	309 [185–452] ^Ψ^	0.35 [0.12–0.80]	4.43 [2.64–7.87]	3.60 [2.80–5.20] ^δ^	14.5 [11.5–17.3]	0.25 [0.20–0.37] ^δ^	1.38 [0.99–1.86]

* *p* vs. CC < 0.050; ** *p* vs. CC ≤ 0.010; ^a^ *p* vs. CC = 0.090; ^b^ *p* vs. CC = 0.111; ^c^ *p* vs. CC = 0.107; ^d^ *p* vs. CC = 0.054; ^e^ *p* vs. CC = 0.057; ^f^ *p* vs. CC = 0.084; ^g^ *p* vs. GG = 0.068; ^h^ *p* vs. GG = 0.113; ^i^ *p* vs. AA = 0.063; ^j^ *p* vs. GG = 0.103; **^δ^** *p* vs. TT < 0.050; ^Ψ^ *p* vs. GG < 0.050; **^λ^** *p* vs. GG < 0.050; ^κ^ *p* vs. AA < 0.050; DV, dialysis vintage; F, female; Hb, hemoglobin; hs-CRP, high-sensitivity C-reactive protein; IL, interleukin; M, male; PTX, pentraxin; sTNFR, soluble tumor necrosis factor receptor. Data are presented as means ± standard deviations or as medians [inter-quartile ranges].

**Table 4:** Age, sex, dialysis vintage, and analytical data, according to the *IL6* (rs1800796, rs1800797, and rs1554606) polymorphic genotypes, for patients with end-stage kidney disease.

	Age (Years)	Sex, F/M (%)	DV (years)	Erythrocyte (×10^12^/L)	Hb (g/dL)	Ferritin (ng/mL)	hs-CRP (mg/dL)	IL6 (pg/mL)	TNF-α (pg/mL)	sTNFR2 (ng/mL)	TNF/TNFR2 (×10^−3^)	PTX3 (ng/mL)
**rs1800796**												
GG (*n* = 247)	69 ± 14	45/55	3.86 [1.68–7.43]	3.75 [3.46–4.01]	11.5 [10.7–12.3]	319 [175–454]	0.41 [0.16–0.81]	4.09 [2.73–7.10] ^a^	3.39 [2.70–4.58]	14.8 [11.9–17.8]	0.24 [0.18–0.31]	1.38 [0.98–2.05]
CC (*n* = 4)	57 ± 24	75/25	3.26 [1.32–23.99]	3.84 [3.48–4.08]	11.8 [10.4–12.4]	280 [173–376]	0.32 [0.05–2.11]	2.51 [1.62–4.55]	2.42 [2.32–6.05]	14.5 [12.8–18.2]	0.18 [0.16–0.33]	1.71 [0.95–2.32]
GC (*n* = 26)	68 ± 12	39/61	4.00 [1.65–7.04]	3.69 [3.45–3.91]	11.4 [10.4–12.4]	270 [209–488]	0.21 [0.11–0.69] ^b^	4.56 [3.34–9.16] ^c^	3.53 [2.53–4.98]	13.3 [11.2–16.5]	0.26 [0.20–0.40]	1.45 [0.85–2.03]
**rs1800797**												
AA (*n* = 31)	70 ± 13	39/61	3.86 [2.07–6.25]	3.70 [3.48–4.01]	11.3 [11.0–12.0]	235 [151–429]	0.50 [0.24–0.89]	4.05 [3.47–7.87]	3.60 [2.70–4.56]	15.2 [12.4–17.8]	0.24 [0.18–0.32]	1.41 [0.95–2.48]
GG (*n* = 135)	69 ± 13	47/53	4.10 [1.98–7.95]	3.80 [3.44–4.06]	11.5 [10.6–12.4]	302 [175–452]	0.29 [0.13–0.79] ^d^	4.39 [2.69–6.94]	3.60 [2.72–5.19]	15.1 [12.0–17.8]	0.25 [0.18–0.36]	1.36 [0.93–2.09]
AG (*n* = 111)	68 ± 15	42/58	3.30 [1.47–7.25]	3.72 [3.46–3.97]	11.4 [10.7–12.0]	322 [190–466] e	0.38 [0.16–0.82]	4.09 [2.58–7.28]	3.08 [2.59–4.20] *	14.0 [11.4–17.4] ^f^	0.23 [0.18–0.29]	1.42 [1.02–1.86]
**rs1554606**												
TT (*n* = 34)	69 ± 13	41/59	4.31 [2.00–6.35]	3.70 [3.48–4.00]	11.4 [11.0–12.1]	244 [154–463]	0.44 [0.20–0.71]	3.98 [3.46–6.86]	3.23 [2.60–4.55]	14.9 [12.3–18.0]	0.24 [0.18–0.29]	1.41 [0.95–2.26]
GG (*n* = 127)	69 ± 13	47/53	4.10 [1.98–8.18]	3.73 [3.43–4.06]	11.4 [10.6–12.4]	302 [183–454]	0.28 [0.13–0.77]	4.23 [2.73–6.69]	3.60 [2.71–5.20]	14.8 [12.0–18.2]	0.25 [0.18–0.37]	1.36 [0.93–2.18]
TG (*n* = 116)	68 ± 15	43/57	3.29 [1.46–7.09]	3.77 [3.49–3.98]	11.6 [10.7–12.1]	320 [185–456] ^δ^	0.41 [0.18–0.88]	4.44 [2.58–7.68]	3.11 [2.66–4.35] ^g^	14.4 [11.4–17.3]	0.23 [0.18–0.29]	1.41 [1.03–1.86]

* *p* vs. GG < 0.050; **^δ^** *p* vs. GG < 0.050; ^a^ *p* vs. CC = 0.104; ^b^ *p* vs. GG = 0.079; ^c^ *p* vs. CC = 0.082; ^d^ *p* vs. AA = 0.119; ^e^ *p* vs. AA = 0.121; ^f^ *p* vs. GG = 0.133; ^g^ *p* vs. GG = 0.061; DV, dialysis vintage; F, female; Hb, hemoglobin; hs-CRP, high-sensitivity C-reactive protein; IL, Interleukin; M, male; PTX, pentraxin; sTNFR, soluble tumor necrosis factor receptor. Data are presented as means ± standard deviations or as medians (inter-quartile ranges).

**Table 5:** Correlations between the inflammatory biomarkers within *TNFRSF1B* rs3397 polymorphic genotypes in patients with end-stage kidney disease (*n* = 277).

	Biomarker vs.	hs-CRP (mg/dL)	*IL6* (pg/mL)	sTNFR2 (ng/mL)	TNF-α/sTNFR2 (×10^−3^)	PTX3 (ng/mL)
CC (*n* = 26)	TNF-α (pg/mL)	0.121	0.229	0.396 *	0.625 ***	−0.126
	IL6 (pg/mL)	0.731 ***	--	−0.009	0.143	−0.212
	sTNFR2 (ng/mL)	0.139	−0.009	--	−0.385	0.073
TT (*n* = 128)	TNF-α (pg/mL)	0.255 **	0.361 ***	0.240 **	0.763 ***	0.158
	IL6 (pg/mL)	0.561 ***	--	0.338 ***	0.153	0.260 **
	sTNFR2 (ng/mL)	0.299 ***	0.338 ***	--	−0.369 ***	0.282 ***
TC (*n* = 123)	TNF-α (pg/mL)	0.227 *	0.153	0.268 **	0.765 ***	−0.025
	IL6 (pg/mL)	0.511 ***	--	0.201 *	0.018	0.096
	sTNFR2 (ng/mL)	0.219 *	0.201 *	--	−0.342 ***	0.084

* *p* < 0.05; ** *p* < 0.01; and *** *p* ≤ 0.001 for correlations between parameters for *TNFR2* rs3397 polymorphic genotypes (Spearman’s rank correlation r). hs-CRP, high-sensitivity C-reactive protein; IL, Interleukin; PTX, pentraxin; sTNFR, soluble tumor necrosis factor receptor.

## Data Availability

Data are contained within the article or Supplementary Material.

## Supplementary Materials

The following supporting information can be downloaded at https://www.mdpi.com/article/10.3390/biomedicines12061228/s1, Figure S1: Two-year survival probability cumulative curves for all-cause mortality for patients with end-stage renal disease (*n* = 250) according to the *TNFRSF1B* (rs3397, rs1061624, and rs1061622) and *IL6* (rs1800796, rs1800797, and rs1554606) single-nucleotide polymorphisms. Survival distribution comparisons between genotypes was performed using the log-rank test; Table S1: Demographic, clinical, and analytical data for the control group (*n* = 32) and patients with end-stage kidney disease (ESKD; *n* = 277); Table S2: Odds ratio used to assess the association between the type of allele and the type of individual (control/patient); Table S3: Genotype distribution of *TNFRSF1B* (rs3397, rs1061624, and rs1061622) and *IL6* (rs1800796, rs1800797, and rs1554606) single-nucleotide polymorphisms in patients with end-stage kidney disease according to their outcomes after a two-year follow-up; Table S4: Two-year estimate of all-cause mortality hazard ratio (HR) for patients with end-stage kidney disease (*n* = 250) according to the *TNFRSF1B* (rs3397, rs1061624, and rs1061622) and *IL6* (rs1800796, rs1800797, and rs1554606) single-nucleotide polymorphisms.
