# Supplementary material for: Impact of TNFRSF1B (rs3397, rs1061624 and rs1061622) and IL6 (rs1800796, rs1800797 and rs1554606) Gene Polymorphisms on Inflammatory Response in Patients with End-Stage Kidney Disease Undergoing Dialysis
Source: Biomedicines. 2024 May 31;12(6):1228. doi: 10.3390/biomedicines12061228 (PMC11200861; doi:10.3390/biomedicines12061228)
Supplement: Supplementary file 1 [file biomedicines-12-01228-s001.zip › biomedicines-3012219-supplementary.pdf]

Supplementary Materials

Table S1. Demographic, clinical, and analytical data in control group (*n* = 32) and patients with end-stage kidney disease (ESKD; *n* = 277).

	**Control**	**ESKD**	***p***
Age (years)	56 ± 5	69 ± 14	**< 0.001**
Sex, F/M (%)	59 / 41	44 / 56	0.108
BMI (kg/m²)	25 ± 3	26 ± 5	0.298
Dialysis vintage (years)	---	3.9 [1.7–7.4]	---
URR (%)	---	79 [76–83]	---
eKt/V	---	1.6 ± 0.3	---
Ultrafiltration volume (L)	---	2.3 ± 0.9	---
CKD Etiology, *n* (%)			
- Diabetic nephropathy	---	96 (34.7)	---
- Hypertensive nephrosclerosis	---	35 (12.6)	---
- Polycystic kidney disease	---	19 (6.9)	---
- Chronic glomerulonephritis	---	23 (8.3)	---
- Other	---	42 (15.2)	---
- Undetermined	---	62 (22.4)	---
Dialysis therapy, *n* (%)			
- Hemodialysis	---	40 (14.4]	---
- On-line hemodiafiltration	---	237 (85.6)	---
Vascular access, *n* (%)			
- Arteriovenous fistula	---	224 (80.9)	---
- Arteriovenous graft	---	14 (5.1)	---
- Central venous catheter	---	39 (14.1)	---
Analytical data:			
- Erythrocyte (x10^{12}/L)	4.59 [4.29–5.04]	3.74 [3.46–4.01]	**< 0.001**
- Hemoglobin (g/dL)	13.8 [13.1–15.6]	11.5 [10.7–12.3]	**< 0.001**
- Ferritin (ng/mL)	88 [44–157]	309 [178–455]	**< 0.001**
- hs-CRP (mg/dL)	0.15 [0.04–0.27]	0.37 [0.16–0.81]	**< 0.001**
- IL6 (pg/mL)	1.15 [0.74–1.62]	4.22 [2.73–7.25]	**< 0.001**
- TNF-α (pg/mL)	0.81 [0.68–1.07]	3.39 [2.67–4.63]	**< 0.001**
- sTNFR2 (ng/mL)	2.0 [1.8–2.4]	14.7 [11.9–17.6]	**< 0.001**
- TNF-α/sTNFR2 (x10^{-3})	0.40 [0.32–0.52]	0.32 [0.18–0.32]	**< 0.001**
- PTX3 (ng/mL)	0.58 [0.42–0.73]	1.39 [0.98–2.06]	**< 0.001**

BMI, body mass index; CKD, chronic kidney disease; F, female; hs-CRP, high-sensitivity C-reactive protein; IL, Interleukin; M, male; PTX, pentraxin; sTNFR, soluble tumor necrosis factor receptor; URR, urea reduction ratio.
Data are presented as mean ± standard deviation or as median [inter-quartile range].

Table S2. Odds ratio to assess the association between the type of allele and the type of individual (Control/Patient).

			**OR for Group (Control/Patient)**	**95% CI**	
TNFRSF1B	rs3397	Allele T	1.911	1.133–3.223	Significant association
	rs1061624	Allele G	0.953	0.566–1.605	No association
	rs1061622	Allele G	1.541	0.762–3.115	No association
IL6	rs1800796	Allele C	0.981	0.336–2.859	No association
	rs1800797	Allele G	0.797	0.445–1.427	No association
	rs1554606	Allele G	0.914	0.523–1.596	No association

CI, Confidence Interval; OR, Odds ratio.

Table S3. Genotype distribution of *TNFRSF1B* (rs3397, rs1061624, and rs1061622) and *IL6* (rs1800796, rs1800797, and rs1554606) single nucleotide polymorphisms in patients with end-stage kidney disease according to their outcome after a two-year follow-up.

	**Alive (*n* = 199)**	**Deceased (*n* = 51)**	***p* (χ^2)**
TNFRSF1B rs3397			
**Genotype (*n*, %)**	Frequency		
CC	16; 8.0%	8; 15.7%	0.143
TT	98; 49.2%	19; 37.3%	
CT	85; 42.7%	24; 47.1%	
TNFRSF1B rs1061624			
**Genotype (*n*, %)**	Frequency		
AA	43; 21.6%	6; 11.8%	0.261
GG	57; 28.6%	18; 35.3%	
AG	99; 49.7%	27; 52.9%	
TNFRSF1B rs1061622			
**Genotype (*n*, %)**	Frequency		
TT	122; 61.3%	34; 66.7%	0.778
GG	13; 6.5%	3; 5.9%	
TG	64; 32.2%	14; 27.5%	
IL6 rs1800796			
**Genotype (*n*, %)**	Frequency		
GG	181; 91.0%	46; 90.2%	0.597
CC	3; 1.5%	0; 0%	
GC	15; 7.5%	5; 9.8%	
IL6 rs1800797			
**Genotype (*n*, %)**	Frequency		
AA	17; 8.5%	7; 13.7%	0.495
GG	97; 48.7%	22; 43.1%	
AG	88; 42.7%	22; 43.1%	
IL6 rs1554606			
**Genotype (*n*, %)**	Frequency		
TT	20; 10.1%	8; 15.7%	0.389
GG	91; 45.7%	19; 37.3%	
TG	88; 44.2%	24; 47.1%	

χ^2, Pearson's Chi-squared test; IL6, interleukin-6.

Table S4. Two-year estimate of all-cause mortality hazard ratio (HR) in patients with end-stage kidney disease (n = 250) according to the *TNFRSF1B* (rs3397, rs1061624, and rs1061622) and *IL6* (rs1800796, rs1800797, and rs1554606) single nucleotide polymorphisms.

SNP	p	HR	95.0% CI for HR
TNFRSF1B rs3397	0.884	1.034	0.662–1.616
TNFRSF1B rs1061624	0.141	1.358	0.904–2.041
TNFRSF1B rs1061622	0.563	0.911	0.665–1.248
IL6 rs1800796	0.374	1.243	0.770–2.007
IL6 rs1800797	0.908	0.975	0.638–1.491
IL6 rs1554606	0.855	1.040	0.685–1.578

The Cox Proportional Hazards Survival Regression model was adjusted for age, dialysis vintage, vascular access, and the co-morbidities, type 2 diabetes and cardiovascular disease; the analysis was performed using as reference the heterozygous genotype patients. CI, confidence interval; IL6, interleukin-6; SNP, single nucleotide polymorphisms.

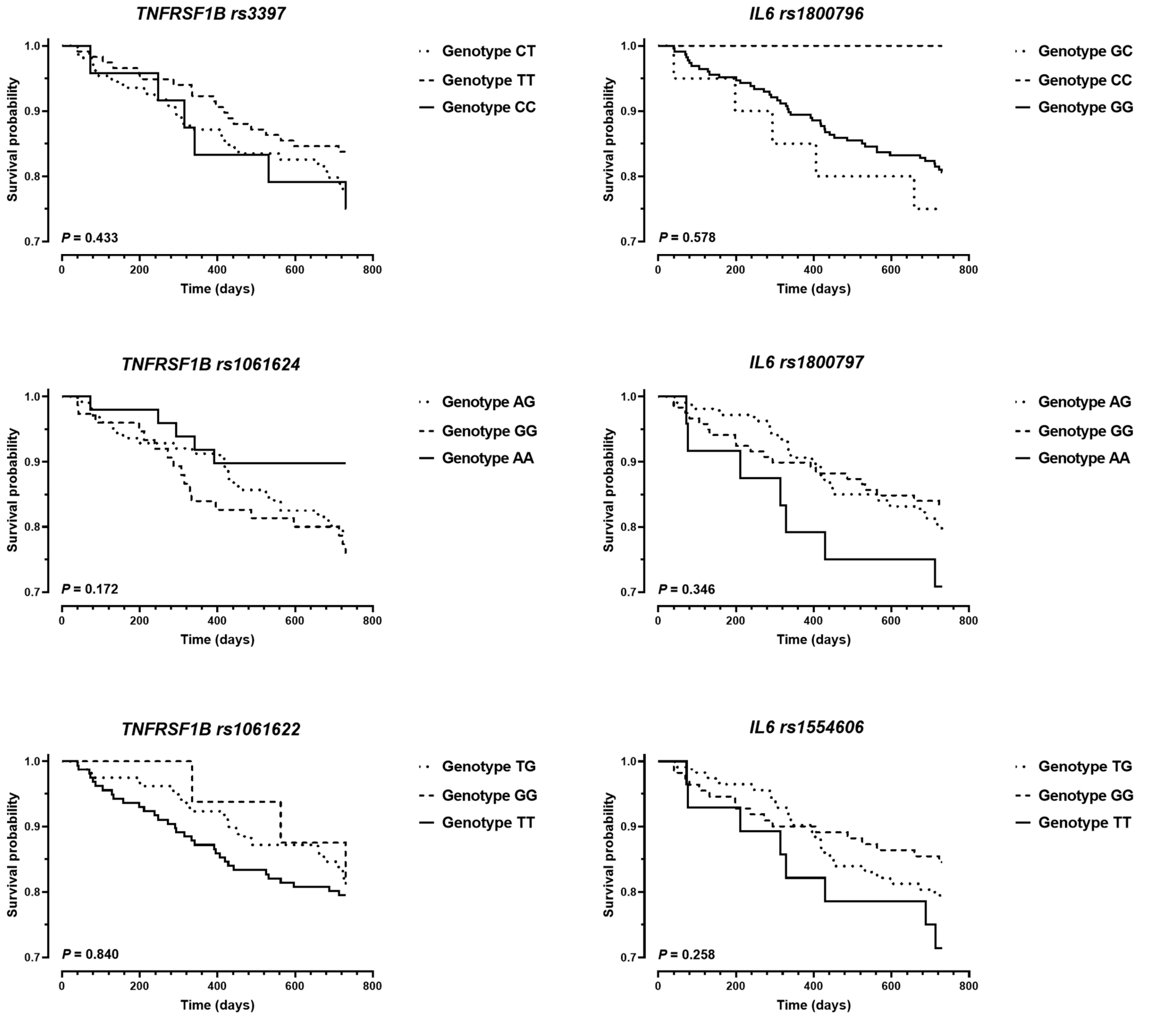

Figure S1. Two-year survival probability cumulative curves for all-cause mortality in patients with end-stage renal disease (n = 250) according to the *TNFRSF1B* (rs3397, rs1061624, and rs1061622) and *IL6* (rs1800796, rs1800797, and rs1554606) single nucleotide polymorphisms. Survival distribution comparisons between genotypes was performed by the log-rank test.
